# The incidence and mortality of lung cancer in China: a trend analysis and comparison with G20 based on the Global Burden of Disease Study 2019

**DOI:** 10.3389/fonc.2023.1177482

**Published:** 2023-08-09

**Authors:** Jianhai Long, Mimi Zhai, Qin Jiang, Jiyang Li, Cixian Xu, Duo Chen

**Affiliations:** ^1^ Department of Pulmonary and Critical Care Medicine, Beijing Tiantan Hospital, Capital Medicine University, Beijing, China; ^2^ Xiangya Nursing School, Central South University, Changsha, Hunan, China; ^3^ Department of General Surgery, The Second Xiangya Hospital, Central South University, Changsha, Hunan, China; ^4^ Public Health Emergency Center, Beijing Center for Disease Prevention and Control, Beijing, China; ^5^ Department of Respiratory and Critical Care Medicine, Beijing Institute of Respiratory Medicine and Beijing Chao-Yang Hospital, Capital Medical University, Beijing, China

**Keywords:** lung cancer, GBD (Global Burden of Disease), China, G20 countries, mortality to incidence ratio

## Abstract

**Background:**

Lung cancer is a significant health concern in China. There is limited available data of its burden and trends. This study aims to evaluate the trends of lung cancer across different age groups and genders in China and the Group of Twenty (G20) countries, explore the risk factors, and predict the future trends over a 20-year period.

**Methods:**

The data were obtained from the GBD study 2019. The number of cases, age standardized rate (ASR), and average annual percentage changes (AAPC) were used to estimate the trend in lung cancer by age, gender, region and risk factor. The trend of lung cancer was predicted by autoregressive integrated moving average (ARIMA) model by the “xtarimau” command. The joinpoint regression analysis was conducted to identify periods with the highest changes in incidence and mortality. Additionally, the relationship between AAPCs and socio-demographic index (SDI) was explored.

**Results:**

From 1990 to 2019, both the incidence and mortality of lung cancer in China and G20 significantly increased, with China experiencing a higher rate of increase. The years with the highest increase in incidence of lung cancer in China were 1998-2004 and 2007-2010. Among the G20 countries, the AAPC in incidence and mortality of lung cancer in the Republic of Korea was the highest, followed closely by China. Although India exhibited similarities, its AAPC in lung cancer incidence and mortality rates was lower than that of China. The prediction showed that the incidence in China will continue to increase. In terms of risk factors, smoking was the leading attributable cause of mortality in all countries, followed by occupational risk and ambient particulate matter pollution. Notably, smoking in China exhibited the largest increase among the G20 countries, with ambient particulate matter pollution ranking second.

**Conclusion:**

Lung cancer is a serious public health concern in China, with smoking and environmental particulate pollution identified as the most important risk factors. The incidence and mortality rates are expected to continue to increase, which places higher demands on China’s lung cancer prevention and control strategies. It is urgent to tailor intervention measures targeting smoking and environmental pollution to contain the burden of lung cancer.

## Introduction

Lung cancer is a highly aggressive and widespread cancer that ranks among the leading causes of cancer-related deaths worldwide (1). Lung cancer caused 1.80 million deaths worldwide in 2020, accounting for approximately one fifth of all cancer deaths and standing as the primary cause of cancer mortality worldwide (2, 3). Since the 1990s, lung cancer has been the leading cause of cancer death in China, responsible for over one third of the global total. Over the past 30 years, half of the global increase in lung cancer deaths has occurred in China (4). Lung cancer can be categorized into small cell lung cancer (SCLC) and non-small cell lung cancer (NSCLC) based on pathological typing. The incident and mortality trend of SCLC and NSCLC vary across countries. It decreased in United States from 2009 to 2018, especially in NSCLC (5, 6). Additionally, the trend of NSCLC and SCLC remained stable in Switzerland (7). However, it demonstrated an increase trend in incidence in female in China (8). Although new treatment strategies, such as targeted therapies that inhibit specific mutated tyrosine kinases or downstream factors (9), immunotherapy that reactivates the immune system to eliminate LC cells (10), novel multi-tier biotechnology treatments (1, 11), and etc (12, 13), have emerged, the success rate of therapy remains relatively low. Moreover, the prognosis for late-stage lung cancer patients is worse than for other types of cancer. Therefore, it is of great significance to analyze the trends of lung cancer in China and its differences with other countries to make health policies and healthcare measures. However, the current epidemiology of lung cancer in China is mainly based on regional data or specific age groups, and the research time span is relatively short, which limits the analysis of lung cancer trends (14–17).

The G20 is an international economic cooperation forum composed of multiple regions including Asia, Africa, and Europe. Distinguishing itself from Organization for Economic Cooperation and Development (OECD) and Asia-Pacific Economic Cooperation (APEC), the G20 encompasses both developed and developing countries, with a characteristic of diversity in its member countries and wide geographical distribution (18). Comparing China’s health challenges with those of the G20 is crucial to clarify the advantages and disadvantages of China’s health performance in lung cancer, and to provide guidance for prevention and control of lung cancer in China.

This study was based on the results of the GBD 2019, and introduced the latest evaluation indicator mortality-to-incidence ratio (MIR). It comprehensively analyzed the trends of the incidence, mortality and MIR of lung cancer by age and gender in China and G20 from 1990 to 2019. The study also explored the correlation between SDI and AAPC. Additionally, the study predicted the trends of incidence and mortality rates of lung cancer in China and the G20 countries over the next 20 years. This study helps to identify the focus and direction of public health work and provides data for health policy decision-makers to allocate scarce medical resources accurately and efficiently.

## Materials and methods

### Case definition and data sources

In GBD 2019, lung cancer was defined as a malignant tumor originating from the mucous membrane of the bronchus or glands in lungs. It was categorized into SCLC and NSCLC, with NSCLC being the predominant pathological type. ICD-10 codes (C34) were used to identify lung cancer cases.

Analysis of lung cancer in China and G20 countries was performed by using data from the GBD database, which includes the global burden of 369 diseases and injuries in 204 countries and territories from 1990 to 2019. The number of cases, incidence, mortality and related risk factors of lung cancer in China and G20 directly extracted from the GBD 2019. This study follows the Guidelines for Accurate and Transparent Health Estimates Reporting (GATHER) for cross-sectional studies.

### SDI

The SDI is a comprehensive measurement of the total fertility rate, lagged distributed income per capita, and mean years of education for individuals over the age of 15 in regions. The SDI is provided by the Institute of Health Metrology and Evaluation. The value of SDI ranged from 0 to 1, where 0 represents the theoretical minimum level of development related to health outcomes and 1 represents the theoretical maximum level.

### MIR

The MIR is an index to compare the disease burden by standardizing mortality to incidence. We calculated the MIR by dividing the Age Standardized Mortality Rate (ASMR) by Age Standardized Incidence Rate (ASIR) for each year from 1990 to 2019 in China and G20 countries.

### Trend prediction

In order to predict the morbidity and mortality of lung cancer, we retrieved the corresponding population data from the United Nations Department of Economics and Social Affairs Population Division stratified by year (1990 to 2040), sex (both genders, male and female) and age. Specific calculation methods of calculating are the same as that reported in previous studies (19). The ARIMA model is a differential integrated moving average autoregressive model, also known as an integrated moving average autoregressive model, which is one of the time series forecasting analysis methods. In ARIMA (p, d, q), AR is “autoregressive,” and p is the number of autoregressive terms. MA is the “moving average,” q is the number of terms in the moving average, and d is the number of differences (order) made to make it a stationary sequence. This study utilized the xtarima command in STATA 17.0 to identify the optimal ARIMA(p,d,q) models to analyze the trend of disease burden based on incidence and mortality, and predicted it from 2020 to 2040.

### Statistical analysis

The ASR and its AAPC were calculated to evaluate the incidence and mortality of lung cancer. All rates were reported per 100000 persons. The 95% uncertainty intervals (UI) were defined by the 25th and 975th values of the 1000 ordered estimates based on the GBD algorithm. A two-tailed *p* value less than 0.05 was considered to be statistically significant.

Joinpoint regression analysis was used to evaluate the long-term trends in incidence and mortality of lung cancer in China and G20 by Joint Command Line. This method described trends by connecting several different line segments on a log scale at “joinpoints”. Analysis started from the minimum number of joinpoints. With more connection points added, the significance of each connection point was tested by using the Monte Carlo permutation method. Age-period-cohort (APC) was calculated to assess trends, and APC was tested to determine whether there was a difference from the null hypothesis of no change. In the final model, each joinpoint reflected a statistically significant change of the trend, and each trend was described by APC (20).

The correlations between AAPC and SDI were evaluated by Pearson correlation analyses to evaluate the potential factors affecting AAPC of ASIR and ASMR. All analyses were conducted with Stata 17. The *p* value less than 0.05 was considered to be statistically significant.

## Results

### Overall trends in incidence, mortality, DALY of lung cancer in China and G20 countries

Overall, the number of lung cancer cases in China increased from 257,040 in 1990 to 833,920 in 2019, with an increase of 224.43% (**Table 1**). During the same period, the number of deaths in China increased by 195.39%, with the deaths of 757,170 in 2019 (**Table 2**). The DALY increased by 146.07% from 6,960,870 in 1990 to 17,128,580 in 2019. The ASIR, ASMR and age standardized DALY rate (ASDR) all increased during the period, with males in China being particularly affected (**Tables 1**, **2**; **S1**). From 1990 to 2019, the AAPC of ASIR was 3.4 (95%UI 3.1 to 3.7), the AAPC of ASMR was 3.1 (95%UI 2.8 to 3.4), and the AAPC of ASDR was 2.5 (95%UI 2.3 to 2.7) (**Tables 1**, **2**; **S1**). It was worth noting that the ASIR of lung cancer in China was similar to that in the G20 in 1990, but in 2019, China’s ASIR increased to 58.56 (95%UI 49.23 to 69.01), which was much higher than that of the G20 (39.85, 95%UI 36.07 to 43.50) (**Table 1**). Similarly, the ASMR in China in 1990 was 21.65 (95%UI 18.68 to 24.88), which was lower than that in the G20 (24.08, 95%UI 22.98 to 25.25) (**Table 2**). However, in 2019, China’s ASMR was 53.23 (95%UI 44.91 to 62.41), which was much higher than that in the G20 (35.46, 95%UI 32.41 to 38.46) (**Table 2**). Additionally, the ASDR of China and G20 was similar in 1990, but it was much higher in China in 2019 with the value of 1204.25 (95%UI 1008.22 to 1422.39) (**Table S1**).

**Table 1:** The incidence cases, age-standardized rates, and temporal trend of lung cancer from 1990 to 2019.

Characteristics			1990		2019		1990–2019
			Incidence casesNo. ×10^3^ (95% UI)	ASIR per 100,000No. (95% UI)	Incidence casesNo. ×10^3^ (95% UI)	ASIR per 100,000No. (95% UI)	AAPCNo. (95% CI)
China	both		257.04(221.29-293.65)	21.72(18.69-24.81)	832.92(700.29-981.63)	58.56(49.23-69.01)	3.40(3.10-3.70)
		5-14 years	0.08(0.07-0.10)	0.04(0.03-0.05)	0.04(0.03-0.05)	0.03(0.02-0.03)	-1.40(-1.80--1.00)
		15-49 years	35.43(30.18-41.23)	5.30(4.51-6.17)	53.18(43.79-63.48)	7.38(6.08-8.81)	1.10(0.90-1.40)
		50-69 years	145.14(122.39-168.08)	94.21(79.45-109.11)	395.96(326.09-475.92)	107.34(88.40-129.02)	0.40(0.20-0.60)
		≥70 years	76.39(67.88-85.53)	199.66(177.43-223.55)	383.74(327.63-441.37)	355.43(303.46-408.81)	2.00(1.60-2.30)
	Male		178.99(145.48-213.93)	29.33(23.84-35.06)	576.19(451.40-709.27)	79.49(62.28-97.85)	3.50(3.30-3.60)
		5-14 years	0.05(0.04-0.07)	0.05(0.03-0.06)	0.02(0.02-0.03)	0.03(0.02-0.04)	-1.70(-2.00--1.50)
		15-49 years	23.48(18.77-28.60)	6.80(5.43-8.28)	34.32(26.38-43.46)	9.29(7.14-11.76)	1.10(0.90-1.40)
		50-69 years	104.69(84.02-127.01)	131.21(105.30-159.18)	280.66(214.05-354.82)	152.12(116.02-192.32)	0.50(0.40-0.70)
		≥70 years	50.77(43.18-58.79)	307.57(261.56-356.16)	261.19(211.38-312.62)	530.43(429.29-634.89)	1.90(1.70-2.10)
	Female		78.05(65.13-91.71)	13.61(11.36-15.99)	256.74(205.68-314.16)	36.81(29.49-45.04)	3.50(3.20-3.80)
		5-14 years	0.03(0.03-0.04)	0.03(0.03-0.04)	0.02(0.01-0.02)	0.03(0.02-0.03)	-0.90(-1.90-0.10)
		15-49 years	11.96(9.53-14.67)	3.70(2.95-4.54)	18.86(14.41-23.88)	5.37(4.11-6.80)	1.40(0.80-2.00)
		50-69 years	40.45(33.05-48.19)	54.47(44.50-64.89)	115.31(90.92-143.39)	62.54(49.31-77.77)	0.40(0.10-0.70)
		≥70 years	25.61(22.18-29.85)	117.76(101.98-137.25)	122.55(99.27-147.83)	208.68(169.04-251.74)	2.00(1.70-2.30)
G20	both		948.38(907.57-992.83)	25.61(24.51-26.82)	1937.91(1754.17-2115.90)	39.85(36.07-43.50)	1.60(1.30-1.80)
		5-14 years	0.19(0.16-0.22)	0.03(0.02-0.03)	0.15(0.13-0.17)	0.02(0.02-0.03)	-0.80(-1.70-0.10)
		15-49 years	86.48(81.00-92.79)	4.46(4.18-4.79)	104.34(93.10-116.25)	4.21(3.75-4.69)	-0.20(-0.40-0.10)
		50-69 years	522.57(497.87-548.54)	100.53(95.78-105.53)	885.30(798.52-973.61)	86.60(78.11-95.24)	-0.50(-0.70--0.40)
		≥70 years	339.13(322.23-352.58)	214.43(203.74-222.93)	948.13(849.91-1029.15)	258.11(231.37-280.16)	0.60(0.50-0.70)
	Male		693.73(658.58-735.22)	37.09(35.21-39.31)	1293.89(1151.08-1445.69)	52.85(47.02-59.05)	1.20(1.00-1.40)
		5-14 years	0.11(0.09-0.14)	0.03(0.02-0.04)	0.08(0.06-0.09)	0.02(0.02-0.03)	-1.40(-2.30--0.50)
		15-49 years	60.39(55.57-66.23)	6.11(5.62-6.70)	65.49(56.34-75.70)	5.17(4.45-5.98)	-0.50(-0.90--0.10)
		50-69 years	397.05(375.17-422.30)	154.82(146.29-164.67)	608.60(533.60-687.81)	121.13(106.20-136.89)	-0.80(-1.00--0.60)
		≥70 years	236.18(225.18-247.21)	374.65(357.21-392.15)	619.72(556.44-680.55)	386.62(347.14-424.58)	0.10(0.00-0.10)
	Female		254.64(240.23-268.66)	13.90(13.11-14.66)	644.02(570.75-715.04)	26.66(23.63-29.60)	2.30(2.10-2.40)
		5-14 years	0.08(0.07-0.09)	0.02(0.02-0.03)	0.07(0.06-0.09)	0.02(0.02-0.03)	0.10(-0.40-0.50)
		15-49 years	26.09(23.53-28.71)	2.75(2.48-3.03)	38.84(33.60-44.19)	3.20(2.77-3.64)	0.60(0.20-1.00)
		50-69 years	125.52(117.96-133.45)	47.67(44.79-50.68)	276.70(246.79-309.60)	53.23(47.48-59.56)	0.30(0.20-0.40)
		≥70 years	102.96(95.47-108.09)	108.24(100.37-113.64)	328.41(282.91-363.88)	158.61(136.64-175.75)	1.30(1.20-1.40)

ASR, age standardized incidence rate; CI, confidence interval; AAPC, Annual percent change; UI, uncertainty interval.

**Table 2:** The death cases, age-standardized rates, and temporal trend of lung cancer from 1990 to 2019.

Characteristics			1990		2019		1990–2019
			Death cases No. ×10^3^ (95% UI)	ASMR per 100,000 No. (95% UI)	Death cases No. ×10^3^ (95% UI)	ASMR per 100,000 No. (95% UI)	AAPC No. (95% CI)
China	both		256.33(221.06-294.56)	21.65(18.68-24.88)	757.17(638.74-887.75)	53.23(44.91-62.41)	3.10(2.80-3.40)
		5-14 years	0.07(0.05-0.08)	0.03(0.03-0.04)	0.03(0.02-0.03)	0.02(0.02-0.02)	-1.80(-2.20--1.40)
		15-49 years	31.64(26.72-37.06)	4.73(4.00-5.54)	41.95(34.76-50.12)	5.82(4.82-6.95)	0.70(0.40-0.90)
		50-69 years	138.20(116.70-161.05)	89.71(75.75-104.54)	329.93(271.99-396.05)	89.44(73.73-107.37)	-0.10(-0.20-0.10)
		≥70 years	86.41(77.09-97.22)	225.87(201.49-254.12)	385.27(328.28-445.70)	356.84(304.06-412.82)	1.60(1.30-1.80)
	Male		177.93(145.87-214.39)	29.16(23.91-35.14)	523.19(413.19-647.41)	72.18(57.01-89.32)	3.20(3.00-3.30)
		5-14 years	0.04(0.03-0.05)	0.04(0.03-0.05)	0.02(0.01-0.02)	0.02(0.02-0.03)	-2.10(-2.40--1.90)
		15-49 years	21.06(16.85-26.16)	6.10(4.88-7.57)	27.62(21.28-35.42)	7.47(5.76-9.58)	0.80(0.50-1.00)
		50-69 years	99.73(80.24-121.68)	124.99(100.57-152.50)	235.76(181.61-297.22)	127.79(98.44-161.10)	0.10(-0.10-0.30)
		≥70 years	57.09(48.67-66.57)	345.85(294.80-403.24)	259.80(210.92-312.45)	527.61(428.36-634.54)	1.50(1.30-1.70)
	Female		78.40(65.03-91.66)	13.67(11.34-15.98)	233.98(189.18-282.75)	33.54(27.12-40.54)	3.10(2.90-3.40)
		5-14 years	0.03(0.02-0.03)	0.03(0.02-0.03)	0.01(0.01-0.01)	0.02(0.01-0.02)	-1.40(-2.30--0.40)
		15-49 years	10.58(8.48-13.05)	3.28(2.62-4.04)	14.33(11.19-18.01)	4.08(3.19-5.13)	0.90(0.10-1.70)
		50-69 years	38.47(31.37-45.43)	51.80(42.25-61.18)	94.17(74.74-117.30)	51.07(40.54-63.62)	-0.10(-0.40-0.20)
		≥70 years	29.32(25.16-34.15)	134.81(115.67-157.00)	125.47(102.20-148.42)	213.65(174.04-252.74)	1.60(1.30-1.90)
G20	both		891.45(851.00-934.88)	24.08(22.98-25.25)	1724.69(1576.26-1870.71)	35.46(32.41-38.46)	1.40(1.20-1.60)
		5-14 years	0.15(0.12-0.17)	0.02(0.02-0.02)	0.11(0.09-0.12)	0.02(0.01-0.02)	-1.00(-1.90--0.10)
		15-49 years	74.11(69.13-79.88)	3.83(3.57-4.12)	82.46(74.00-91.80)	3.33(2.98-3.70)	-0.40(-0.70--0.20)
		50-69 years	467.26(444.19-492.64)	89.89(85.45-94.78)	727.92(663.25-798.03)	71.21(64.88-78.07)	-0.80(-0.90--0.70)
		≥70 years	349.93(330.83-365.43)	221.26(209.18-231.06)	914.20(824.61-985.88)	248.87(224.48-268.39)	0.40(0.30-0.50)
	Male		654.85(619.51-695.84)	35.01(33.12-37.20)	1159.61(1043.00-1286.52)	47.37(42.61-52.55)	1.00(0.80-1.20)
		5-14 years	0.09(0.07-0.11)	0.02(0.02-0.03)	0.06(0.05-0.07)	0.02(0.01-0.02)	-1.60(-2.50--0.70)
		15-49 years	52.30(48.01-57.78)	5.29(4.85-5.84)	53.00(45.86-60.84)	4.19(3.62-4.81)	-0.80(-1.10--0.40)
		50-69 years	358.08(336.92-382.21)	139.62(131.37-149.03)	509.42(450.74-573.11)	101.39(89.71-114.07)	-1.10(-1.20--0.90)
		≥70 years	244.38(231.78-256.56)	387.66(367.68-406.99)	597.13(539.26-652.47)	372.53(336.42-407.05)	-0.20(-0.20--0.10)
	Female		236.60(221.10-251.36)	12.91(12.07-13.72)	565.08(503.87-624.07)	23.39(20.86-25.84)	2.10(2.00-2.10)
		5-14 years	0.06(0.05-0.07)	0.02(0.01-0.02)	0.05(0.04-0.06)	0.02(0.01-0.02)	-0.20(-0.60-0.30)
		15-49 years	21.81(19.59-24.40)	2.30(2.07-2.57)	29.46(25.71-33.61)	2.43(2.12-2.77)	0.30(-0.10-0.70)
		50-69 years	109.18(101.56-117.09)	41.46(38.57-44.46)	218.50(197.33-243.55)	42.03(37.96-46.85)	0.10(-0.10-0.20)
		≥70 years	105.55(97.29-111.39)	110.97(102.29-117.11)	317.07(271.57-350.72)	153.14(131.16-169.39)	1.10(1.00-1.20)

### Age composition ratio for incidence and mortality of lung cancer

**Figure 1:**
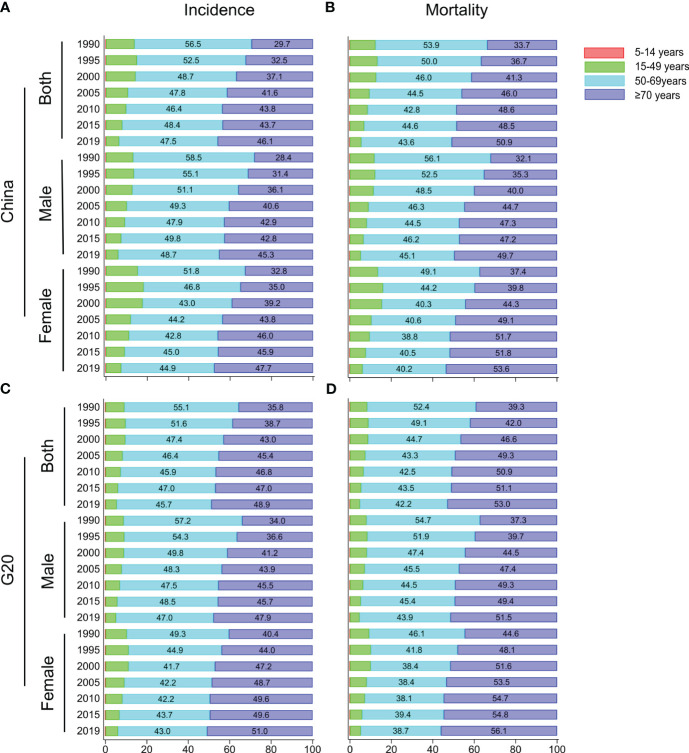
Age distribution trends for incidence and mortality of lung cancer in China and G20 countries. Age distribution of incidence **(A)** and mortality **(B)** of lung cancer for males, females, and both sexes in China from 1990 to 2019. Age distribution of incidence **(C)** and mortality **(D)** of lung cancer for males, females, and both sexes in G20 from 1990 to 2019.

The distribution for age composition ratio of incidence and mortality was similar in **Figures 1A, B** and **Figures 1C, D**. The age group of 50 to 69 and over 70 accounted for more than 80% of all lung cancer cases. In 1990, the age group with the highest proportion of incidence and mortality was the age group of 50 to 69 (**Figures 1A–D**). Over time, the proportion of incidence and mortality for this age group decreased, and increased in the group with age over 70 years old. In 2019, there was a significant reduction in the proportion between the two age groups, and the mortality of lung cancer even exceeded that of the 50-69 age group (**Figure 1A**). It is worth noting that the proportion of the age group of over 70 years old in the G20 countries was higher than that in China in both incidence and mortality, while the age group of 50-69 in China had a higher proportion of incidence and mortality than that in the G20 countries (**Figures 1A–D**).

### Trends in ASIR, ASMR and MIR of lung cancer

**Figure 2:**
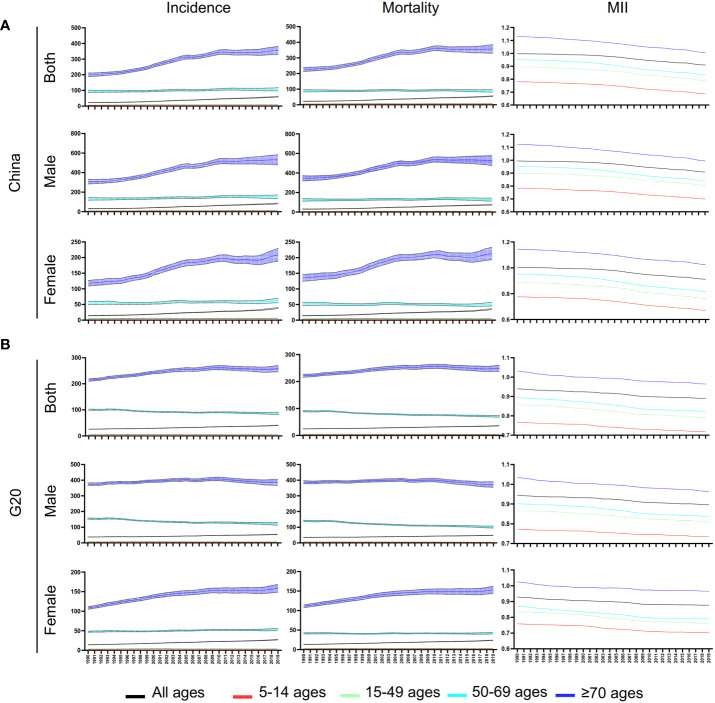
Trends of ASIR, ASMR, and ASR of MIR for lung cancer in China and G20. **(A)** Trends of ASIR, ASMR, and ASR of MIR for lung cancer at different ages for males, females, and both sexes in China from 1990 to 2019. **(B)** Trends of ASIR, ASMR, and ASR of MIR for lung cancer at different ages for males, females, and both sexes in G20 countries from 1990 to 2019.

From 1990 to 2019, ASIR and ASMR in China and G20 showed an upward trend (**Figures 2A, B**). The age group over 70 had the highest ASIR and the most significant upward trend (**Figure 2A**). It is worth noting that the lung cancer ASIR of the 50-69 age group remained stable in China, while the ASIR of lung cancer in the G20 countries showed a downward trend (**Figures 2A, B**). In addition, the ASMR of Chinese males in the over 70 age group showed an upward trend, while it showed a downward trend in the G20 (**Figure 2B**; **Table 2**).

Unlike ASIR and ASMR, the ASR of MIR showed a downward trend both in China and G20 in both sexes and all age groups (**Table 3**; **Figures 2A, B**). The most significant decrease in ASR of MIR was found in the 50-69 years old age group in China (**Table 3**).

**Table 3:** The MIR and temporal trend of lung cancer from 1990 to 2019.

Characteristics			1990	2019	1990–2019
			MIR	MIR	AAPCNo. (95% CI)
China	both		1.00	0.91	-0.30(-0.30--0.30)
		5-14 years	0.78	0.69	-0.40(-0.50--0.40)
		15-49 years	0.89	0.79	-0.40(-0.50--0.40)
		50-69 years	0.95	0.83	-0.50(-0.50--0.40)
		≥70 years	1.13	1.00	-0.40(-0.40--0.40)
	Male		0.99	0.91	-0.30(-0.30--0.30)
		5-14 years	0.78	0.70	-0.40(-0.40--0.40)
		15-49 years	0.90	0.80	-0.40(-0.40--0.40)
		50-69 years	0.95	0.84	-0.40(-0.50--0.40)
		≥70 years	1.12	0.99	-0.40(-0.40--0.40)
	Female		1.00	0.91	-0.30(-0.40--0.30)
		5-14 years	0.78	0.67	-0.50(-0.50--0.50)
		15-49 years	0.88	0.76	-0.50(-0.60--0.50)
		50-69 years	0.95	0.82	-0.50(-0.50--0.50)
		≥70 years	1.14	1.02	-0.40(-0.40--0.40)
G20	both		0.94	0.89	-0.20(-0.20--0.20)
		5-14 years	0.77	0.72	-0.20(-0.20--0.20)
		15-49 years	0.86	0.79	-0.30(-0.30--0.30)
		50-69 years	0.89	0.82	-0.30(-0.30--0.30)
		≥70 years	1.03	0.96	-0.20(-0.30--0.20)
Male			0.94	0.90	-0.20(-0.20--0.10)
		5-14 years	0.77	0.73	-0.20(-0.20--0.10)
		15-49 years	0.87	0.81	-0.20(-0.20--0.20)
		50-69 years	0.90	0.84	-0.30(-0.30--0.20)
		≥70 years	1.03	0.96	-0.20(-0.30--0.20)
Female			0.93	0.88	-0.20(-0.20--0.20)
		5-14 years	0.76	0.70	-0.30(-0.30--0.20)
		15-49 years	0.84	0.76	-0.30(-0.40--0.30)
		50-69 years	0.87	0.79	-0.30(-0.40--0.30)
		≥70 years	1.03	0.97	-0.20(-0.20--0.20)

CI, confidence interval; AAPC, Annual percent change; MIR, mortality to incidence ratio.

### Joinpoint regression analysis of lung cancer incidence, mortality and MIR

**Figure 3:**
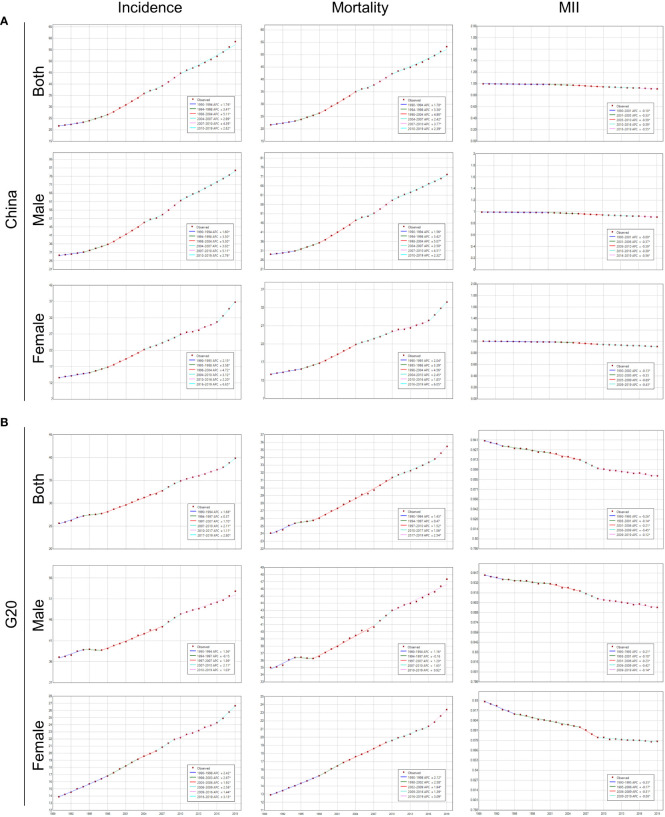
Joinpoint regression analysis of ASIR, ASMR, and MIR of lung cancer in China and G20. **(A)** Joinpoint regression analysis of ASIR, ASMR, and ASR of MIR for lung cancer at different ages for males, females, and both sexes in China from 1990 to 2019. **(B)** Joinpoint regression analysis of ASIR, ASMR, and ASR of MIR for lung cancer at different ages for males, females, and both sexes in G20 from 1990 to 2019.

Between 1990 and 2019, both China and G20 countries showed an increase in lung cancer incidence and mortality, with China experiencing a faster increase (**Tables 1**, **2**; **Figures 3A, B**). The joinpoint regression analysis identified the substantial change in lung cancer incidence in China in 1994, 1998, 2004, 2007 and 2010, and in G20 in 1994, 1997, 2007, 2010 and 2017 (**Figures 3A, B**). The periods with the largest increase in lung cancer incidence in China were from 1998 to 2004 and from 2007 to 2010 (**Figure 3A**). However, the period with the largest increase in lung cancer incidence in the G20 lagged behind that in China, and was from 2017 to 2019 (**Figure 3B**). In addition, the period with the slowest increase in lung cancer incidence in the G20 also lagged behind that in China (**Figures 3A, B**). The period with the slowest increase in lung cancer incidence in Chinese females was from 1990 to 1995, and the period with the slowest increase in mortality was from 2010 to 2016 (**Figure 3A**), while in the G20, it was from 2009 to 2016 (**Figure 3B**).

The substantial changes in lung cancer mortality in China were in 1994, 1998, 2004, 2007 and 2010, and in G20 in 1994, 1997, 2010 and 2017 (**Figures 3A, B**). The trend of mortality was similar to that of incidence in China and G20 (**Figures 3A, B**).

The substantial changes in MIR of lung cancer in China were in 2001, 2005, 2010 and 2016, and in G20 in 1993, 2001, 2006, and 2009 (**Figures 3A, B**). Fortunately, from 1990 to 2019, the MIR showed a downward trend both in China and G20 (**Figures 3A, B**). In 2019, the MIR in the G20 was less than 1, however, the MIR of Chinese females of the age group over 70 was 1.02.

### Trends in risk factors of lung cancer

**Figure 4:**
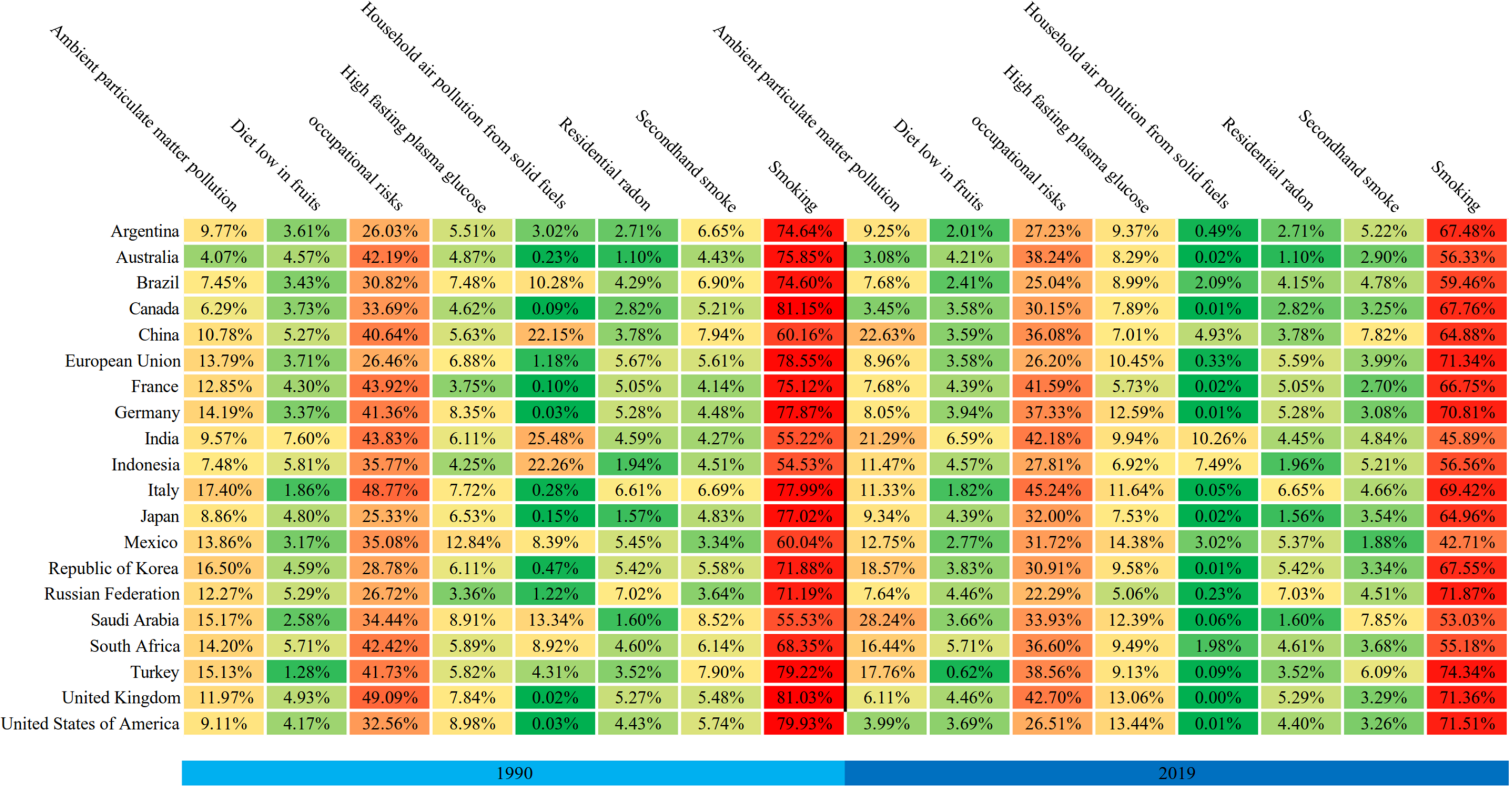
Trends in attributable risk factors for lung cancer deaths in G20 countries in 1990 and 2019.

**Figure 4** showed the risk factors for the G20 in 1990 and 2019. Smoking remained the most significant risk factor in every country, followed by occupational risks and ambient particulate matter pollution. Interestingly, our study found that the number of lung cancer deaths caused by fasting plasma glucose increased in all countries. The three countries with the largest number of deaths attributed to fasting plasma glucose were Mexico, United States of America and the United Kingdom in 2019. Compared to other G20 countries, China’s attributed death caused by fruit intake, occupational risks, and household air pollution from solid fuels had all decreased over the observation period, among which the household air pollution decreased by 17.22%. However, it is worth noting that in 1990, China’s attributed deaths of lung cancer caused by smoking were relatively low, ranking sixteenth among G20. In 2019, only three countries showed an increase trend in attributed deaths caused by smoking. China, however, had the highest increase. Furthermore, China experienced an increase of 22.63% in attributed deaths caused by ambient particulate matter pollution, ranking second after Saudi Arabia.

### Predictions of ASIR and ASMR of lung cancer from 2020 to 2040

**Figure 5:**
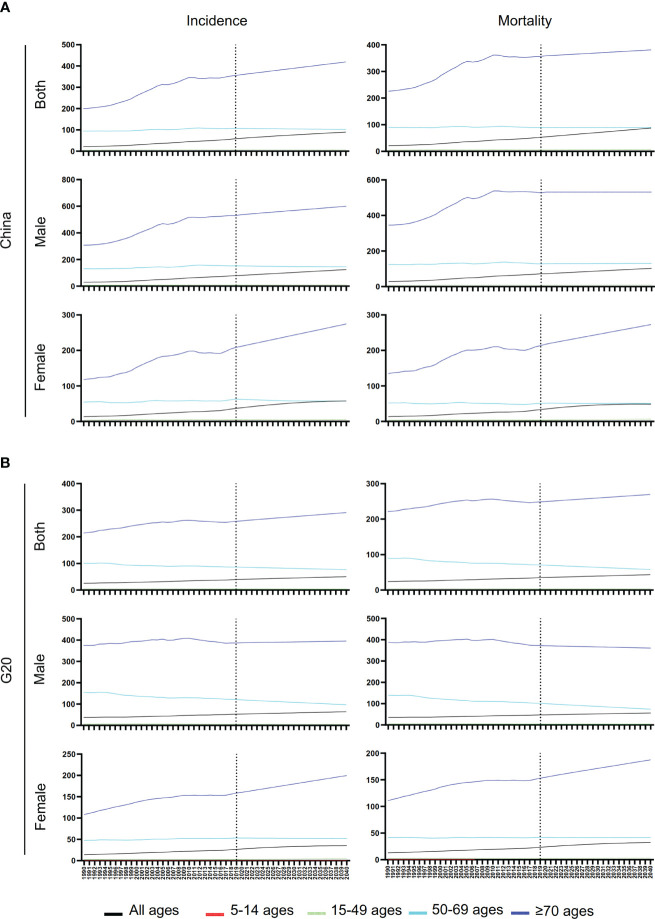
Prediction of ASIR and ASDR of lung cancer in China and G20. **(A)** Prediction of ASIR and ASDR for lung cancer at different ages for males, females, and both sexes in China. **(B)** Prediction of ASIR and ASDR for lung cancer at different ages for males, females, and both sexes in G20.

In 2040, the ASIR of lung cancer in China was predicted to increase to 89.55 per 100,000 persons, with a 52.92% increase from 2019 (**Table 4**; **Figure 5**). Fortunately, the ASIR remained stable except for the over 70 years old age group (**Figure 5A**). In terms of gender, the increase in ASIR was more significant for females in the over 70 years old age group (**Figure 5A**). The ASIR in the G20 also increased, with an increase of 26.47% compared with that in 2019 (**Table 4**; **Figure 5**). Similar gender trends were observed in the G20 countries, but it decreased more significantly in male of the 50-69 age group than in China, with a 20.04% decrease compared to a 4.56% decrease. Compared with the G20, China’s ASMR increased in most age groups during the period. The most significant increase of ASMR was found in female of the over 70 years old age group, with a 27.68% increase (**Figure 5**).

**Table 4:** The prediction of mortality and incidence ASR of lung cancer in 2040.

Characteristics			2040	
			ASIR per 100,000 No.	ASMR per 100,000 No.
China	both		89.55	87.35
		5-14 years	0.04	0.03
		15-49 years	6.64	6.65
		50-69 years	102.67	89.57
		≥70 years	419.24	380.80
	Male		124.45	102.82
		5-14 years	0.04	0.03
		15-49 years	11.11	8.49
		50-69 years	145.19	130.45
		≥70 years	600.23	531.22
	Female		57.63	48.35
		5-14 years	0.03	0.02
		15-49 years	5.06	6.27
		50-69 years	57.37	50.74
		≥70 years	274.56	272.79
G20	both		50.40	43.55
		5-14 years	0.02	0.02
		15-49 years	3.78	3.52
		50-69 years	76.78	57.97
		≥70 years	291.05	269.52
	Male		64.10	56.03
		5-14 years	0.03	0.02
		15-49 years	4.62	3.54
		50-69 years	96.85	73.83
		≥70 years	395.64	360.99
	Female		35.02	32.27
		5-14 years	0.02	0.02
		15-49 years	4.45	3.26
		50-69 years	51.98	41.40
		≥70 years	199.78	187.46

ASIR, age standardized incidence rate, ASMR, ASIR, age standardized mortality rate.

### Association between AAPC and SDI

**Figure 6:**
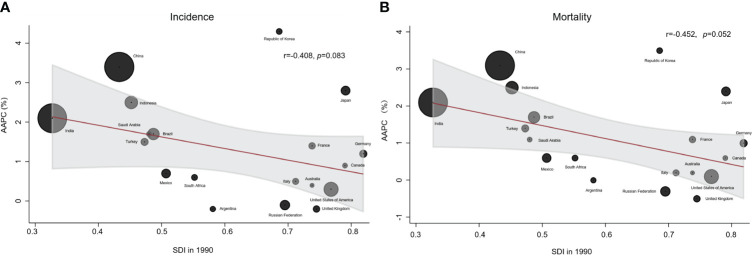
Correlation between AAPC and SDI of ASIR and ASDR for lung cancer. **(A)** Correlation between AAPC of ASIR in 2019 and SDI in 1990. **(B)** Correlation between AAPC of ASMR in 2019 and SDI in 1990.

We found a decreasing trend in AAPC of lung cancer incidence as the SDI increased, although the correlation was not significant (r=-0.408, p=0.083) (**Figure 6A**). Additionally, there was a negative correlation between mortality of AAPC and SDI in 1990, with an almost marginally significant correlation (r=-0.452, p=0.052) (**Figure 6B**).

The AAPC of lung cancer incidence and mortality in the Republic of Korea ranks first among the G20 countries, followed closely by China (**Figure 6**). Notably, India and China shared several similarities, but India’s AAPC for lung cancer incidence and mortality were lower compared to China.

## Discussion

Lung cancer is a major public health issue in the world, especially in China. In the past two decades, lung cancer has become the leading cause of cancer death (21). It is very important to comprehensively evaluate the long-term trends of lung cancer in China and compare it with other countries to establish effective prevention and intervention measures for lung cancer in China. To our knowledge, our study is the first to describe and predict the trend of lung cancer in different age groups and genders from 1990 to 2019 in G20 and China.

Despite efforts were made to control lung cancer from 1990 to 2019, the incidence and mortality continued to increase in China and the G20 countries, and the trend observed in China was particularly worrying. This trend may be related to smoking, air quality and genetic factors.

The primary cause of the rising incidence and mortality of lung cancer in males is the tobacco consumption. Studies indicated that the smoking rate among Chinese men remained high at 60% (22). Unfortunately, the smoking rate among women is rapidly rising (23). It is worth noting that the proportion of teenagers who smoke is also rising. Data from the China CDC shows that the smoking rate among vocational high school students was as high as 21.2% in 2021. In addition, more than one-third of children and non-smokers are exposed to smoking environments (24). Although the Chinese government has formulated a number of policies for smoking cessation, the effects were very limited. Moreover, about 90% of Chinese smokers have not received effective smoking cessation treatment, and the rate of continuous smoking cessation is still low (25). Smokers in China also have a low willingness to quit smoking, and only 16.1% of smokers planning or considering quitting (22). In addition, the awareness and consultation rate for smoking cessation clinics and hotlines in China are very low. At the same time, the proportion of doctors who help patients to quit smoking is only 50% of that in developed countries (26). In contrast to China, the G20 countries have implemented strict tobacco control legislation to reduce smoking rate, resulting in a significant and continuous decrease in per capita tobacco consumption in most countries such as the United States, Japan and Brazil. Especially Australia has enacted smoke-free laws in all states and regions, increased taxes on tobacco products, health warnings on tobacco products, a ban on tobacco advertising, and e-cigarettes. These efforts have led to a significant and continuous decrease in smoking rate in Australia, from 43.4% in 2001 to 12.8% in 2017 (27). Therefore, the Chinese government needs to formulate effective tobacco control policies, such as increasing tobacco taxes, using graphic and text warnings on tobacco product packaging, to effectively reduce the health hazards of smoking.

Although the smoking rate in women was low, the incidence and mortality of lung cancer among Chinese women were higher than those in the G20. This can be attributed to various risk factors, such as ambient particulate matter pollution, household air pollution, and genetics. Over the past decades, China’s rapid economic development, accelerated industrialization and urbanization have led to an increase in energy consumption and industrial waste emissions, resulting in ambient particulate matter pollution in China far higher than in other G20 countries (28). China has been one of the countries with the most severe ambient particulate matter pollution in the world, especially in first-tier cities (29, 30). Although the implementation of the environmental protection policy “Action Plan for the Prevention and Control of Air Pollution” in 2013 led to some improvement in the environment and a decrease in the concentration of PM 2.5 in China from 67.4μg/m^3^ to 45.5μg/m^3^ in 2017 (31), the level is still significantly higher than in some G20 countries, such as Australia (average daily concentration of PM2.5: 12.1–21.7 μg/m3). According to the Ministry of Ecology and Environment of China, 239 out of 338 urban areas (70.7%) suffered from serious air pollution in 2017 (32). In addition, some G20 countries have implemented effective policies to reduce vehicle exhaust and power generation emissions and to promote the development of clean energy (33). Therefore, the government should implement more stringent environmental protection policies, reduce industrial energy consumption and develop clean energy. On the other hand, the household air pollution was another important risk factor for lung cancer among women in China. Coal and other materials have been the main fuel for heating and cooking in China. The World Health Organization (WHO) estimated that approximately 3% of the 5 billion people who were exposed to household air pollution lived in China and India (34, 35). Therefore, clean energy, such as liquefied petroleum gas, natural gas, should be popularized in daily life. Meanwhile, improving the ventilation system in households, changing cooking and eating habits were also very important. In addition, inherited genetic mutations can also contribute to the development of lung cancer, and certain genetic mutations may be more common in the Chinese population, especially among women. Studies have shown that EGFR mutations are the most common genetic aberrations in Chinese NSCLC patients, with a higher frequency than other populations, and the EGFR gene is highly mutated in non-smoking women (36).

In joinpoint analysis, we found that the ASIR of lung cancer had two periods of rapid increase (from 1998 to 2004 and from 2007 to 2010) during the past 30 years. This might be related to the increasing awareness of lung cancer induced by the improvement of health care system. Since the 1990s, China has established three important healthcare systems (37). In 1998, urban employees were included in the basic healthcare system. In addition, regular physical examinations were also gradually being promoted. In 2007, urban residents were included in the basic healthcare system. In 2009, a healthcare system covering most of the population in China was basically established. In 2010, a large-scale, population-based and organized lung cancer screening program was launched nationwide. Therefore, the improvement of people’s awareness of lung cancer and health care system have a greater impact on the incidence and mortality.

In terms of countries, the AAPC of lung cancer incidence and mortality in the Republic of Korea ranked the first among the G20 countries, followed closely by China. Previous studies showed that lung cancer was one of the most common cancers in the Republic of Korea, accounting for 11.7% of new cancer diagnoses (38). This may be closely related to smoking. Although the Republic of Korea government has formulated a number of tobacco control policies, the increasing use of e-cigarette, especially among young people, posed new challenges to lung cancer prevention (39). Secondly, in 2016, the Republic of Korea launched a two-year lung cancer screening project (K-LUCAS), which might contribute to the increased incidence of lung cancer (40). Finally, the aging population also might aggravate the incidence and mortality of lung cancer. Unlike Korea, India, as one of the BRICS countries, had a lower incidence of lung cancer than those of China, although its national conditions were similar to China. This was related to the decline in smoking rates. India was the second-largest consumer and third-largest producer of tobacco in the world. In order to reduce tobacco production and consumption and to raise awareness on the harm of smoking, the National Tobacco Control Program and Prohibition of smoking in public places rules were launched by the government of India (41). Moreover, the Indian government also promulgated the Cigarettes and Other Tobacco Products Act to promote smoking cessation and implementation of the WHO Framework Convention on Tobacco Control (42). According to the Global Adult Tobacco Surveys, smoking consumption had dropped significantly in India (43). Our study also indicated that the proportion of deaths attributable to smoking decreased from 55.22% in 1990 to 45.89% in 2019. Interestingly, our study found that the countries with the fastest increase in incidence and mortality over the past thirty years were the Republic of Korea, China, Japan, and Indonesia, all of which were located in Asia. This may be related to genetic polymorphism and susceptibility to lung cancer in the Asian population.

By predicting the incidence and mortality of lung cancer, we found that the incidence rate in China will continue to rise, which may be related to the future decline in birth rate, the expansion of the proportion of the 65-year-old population, and the further aging of the population structure. Fortunately, the incidence rate in the 50-69 age group will decrease. This may be related to the continuous improvement of the health care system and environmental pollution.

There are some limitations in this study. Firstly, although the GBD study uses as many data sources as possible, data in some countries are still scarce, which may affect the accuracy of the disease burden estimation. Secondly, the time trend of lung cancer may be affected by changes in diagnostic technology over time. Thirdly, this study relies entirely on the GBD database. There are other global databases, such as the WHO’s global health estimates. Fourthly, lung cancer can be classified into SCLC and NSCLC. However, the GBD data only has data on lung cancer and not on the various pathological types. It is not possible to study the trend of SCLC and NSCLC. Finally, based on the GBD database, it is not possible to study the impact of multiple risk factors on lung cancer.

## Conclusion

Our study used the MIR based on the latest GBD data of lung cancer to analyze the trend by ages, regions and genders. In addition, the trend in the next 20 years was predicted by using the latest GBD data of lung cancer in China and G20 countries. Thus, the results could provide data support for early development of prevention and control measures. The joinpoint regression analysis was used to explore the significant change of the trend. By analyzing the joinpoint, we can evaluate the rationality and effectiveness of prevention and control measures and policies. Additionally, by comparing the differences of the trend between China and the G20 countries, it helps us to formulate more reasonable and effective policies and systems to reduce the incidence and mortality of lung cancer.

Our study on the trends and risk factors of lung cancer in incidence and mortality in China and G20 countries showed that the incidence and mortality in China are the highest among G20 countries, and the AAPC is also much higher than the average level of G20 countries. Smoking and ambient particulate matter pollution are the most important risk factors for lung cancer in China, and they are increasing. In the next twenty years, the incidence and mortality rates of lung cancer will continue to rise. Thus, China urgently needs customized interventions against smoking and environmental pollution to curb the burden of lung cancer.

## Data availability statement

Publicly available datasets were analyzed in this study. This data can be found here: http://www.healthdata.org/gbd.

## Author contributions

Study design: JHL, CX and DC. Data collection: MZ, QJ and JYL. Data analysis: JHL, MZ and DC. Figures: QJ and CX. Manuscript writing: JHL, CX and DC. Manuscript proofing: MZ and JYL. All authors contributed to the article and approved the submitted version.
