# Supplementary material for: The incidence and mortality of lung cancer in China: a trend analysis and comparison with G20 based on the Global Burden of Disease Study 2019
Source: Front Oncol. 2023 Aug 9;13:1177482. doi: 10.3389/fonc.2023.1177482 (PMC10446846; doi:10.3389/fonc.2023.1177482)
Supplement: Supplementary file 1 [file Table_1.docx]

TableS1. The DALYs cases, age-standardized rates, and temporal trend of lung cancer from 1990 to 2019

| Characteristics | | | 1990 | |  | 2019 | |  | 1990–2019 |
| --- | --- | --- | --- | --- | --- | --- | --- | --- | --- |
|  |  |  | DALYs | ASDR per 100,000 |  | DALYs | ASDR per 100,000 |  | AAPC |
|  |  |  | No. ×10^3^ (95% UI) | No. (95% UI) |  | No. ×10^3^ (95% UI) | No. (95% UI) |  | No. (95% UI) |
| China | both |  | 6960.87(5966.96-8039.13) | 588.07(504.10-679.16) |  | 17128.58(14340.49-20231.34) | 1204.25(1008.22-1422.39) |  | 2.50(2.30-2.70) |
|  |  | 5-14 years | 5.06(4.03-6.21) | 2.44(1.94-2.99) |  | 2.11(1.73-2.57) | 1.47(1.21-1.79) |  | -1.80(-2.20--1.40) |
|  |  | 15-49 years | 1523.70(1289.87-1779.65) | 227.94(192.96-266.23) |  | 1947.84(1620.99-2333.46) | 270.26(224.91-323.77) |  | 0.50(0.20-0.80) |
|  |  | 50-69 years | 4043.44(3405.68-4719.00) | 2624.71(2210.72-3063.24) |  | 9435.24(7763.00-11312.60) | 2557.79(2104.47-3066.72) |  | -0.10(-0.30-0.10) |
|  |  | ≥70 years | 1388.67(1231.59-1568.26) | 3629.74(3219.15-4099.13) |  | 5743.40(4879.75-6681.09) | 5319.70(4519.75-6188.21) |  | 1.30(1.10-1.60) |
|  | Male |  | 4864.23(3954.89-5914.77) | 797.17(648.14-969.34) |  | 11967.77(9374.37-14952.33) | 1651.13(1293.33-2062.90) |  | 2.50(2.40-2.70) |
|  |  | 5-14 years | 3.10(2.24-4.03) | 2.88(2.08-3.74) |  | 1.21(0.91-1.53) | 1.55(1.17-1.97) |  | -2.10(-2.40--1.80) |
|  |  | 15-49 years | 1008.93(810.96-1248.09) | 292.07(234.76-361.31) |  | 1278.44(990.65-1631.01) | 345.87(268.01-441.26) |  | 0.60(0.40-0.80) |
|  |  | 50-69 years | 2920.83(2349.42-3571.00) | 3660.66(2944.51-4475.51) |  | 6737.51(5185.97-8499.06) | 3651.84(2810.88-4606.64) |  | 0.00(-0.20-0.20) |
|  |  | ≥70 years | 931.37(792.28-1090.91) | 5641.95(4799.39-6608.44) |  | 3950.62(3193.62-4767.77) | 8023.20(6485.83-9682.74) |  | 1.20(1.00-1.40) |
|  | Female |  | 2096.64(1730.33-2468.93) | 365.59(301.72-430.51) |  | 5160.82(4138.83-6353.14) | 739.87(593.36-910.81) |  | 2.50(2.20-2.70) |
|  |  | 5-14 years | 1.96(1.58-2.43) | 1.96(1.58-2.43) |  | 0.90(0.70-1.15) | 1.37(1.06-1.75) |  | -1.40(-2.40--0.40) |
|  |  | 15-49 years | 514.77(412.65-634.40) | 159.36(127.75-196.39) |  | 669.40(524.55-839.07) | 190.66(149.40-238.98) |  | 0.60(-0.30-1.50) |
|  |  | 50-69 years | 1122.60(914.03-1324.88) | 1511.66(1230.81-1784.04) |  | 2697.73(2138.60-3371.02) | 1463.09(1159.85-1828.24) |  | -0.20(-0.40-0.10) |
|  |  | ≥70 years | 457.31(391.57-533.13) | 2102.53(1800.30-2451.12) |  | 1792.79(1469.71-2115.83) | 3052.85(2502.71-3602.95) |  | 1.30(1.00-1.60) |
|  |  |  |  |  |  |  |  |  |  |
| G20 | both |  | 22439.32(21300.07-23632.75) | 606.06(575.29-638.29) |  | 37877.36(34643.06-41264.25) | 778.79(712.29-848.43) |  | 0.90(0.70-1.00) |
|  |  | 5-14 years | 11.28(9.44-12.94) | 1.59(1.33-1.83) |  | 8.26(7.12-9.45) | 1.23(1.06-1.41) |  | -0.90(-1.60--0.20) |
|  |  | 15-49 years | 3482.81(3248.09-3752.90) | 179.76(167.64-193.70) |  | 3819.05(3431.85-4240.86) | 154.01(138.40-171.03) |  | -0.50(-0.70--0.30) |
|  |  | 50-69 years | 13527.76(12844.98-14271.69) | 2602.49(2471.13-2745.60) |  | 20783.40(18947.36-22751.84) | 2033.11(1853.51-2225.67) |  | -0.90(-1.00--0.70) |
|  |  | ≥70 years | 5417.47(5149.04-5659.62) | 3425.39(3255.67-3578.50) |  | 13266.65(12124.85-14307.70) | 3611.57(3300.74-3894.97) |  | 0.20(0.10-0.30) |
|  | Male |  | 16667.45(15682.37-17814.60) | 891.11(838.45-952.45) |  | 25832.19(23013.59-28828.20) | 1055.21(940.07-1177.59) |  | 0.60(0.40-0.70) |
|  |  | 5-14 years | 6.78(5.40-8.26) | 1.86(1.48-2.26) |  | 4.25(3.48-5.12) | 1.21(0.99-1.46) |  | -1.60(-2.50--0.60) |
|  |  | 15-49 years | 2442.34(2237.66-2703.02) | 246.93(226.23-273.28) |  | 2446.03(2117.77-2806.26) | 193.26(167.33-221.73) |  | -0.80(-1.10--0.50) |
|  |  | 50-69 years | 10386.51(9772.06-11110.98) | 4049.94(3810.35-4332.43) |  | 14522.25(12831.54-16367.03) | 2890.36(2553.86-3257.52) |  | -1.10(-1.30--1.00) |
|  |  | ≥70 years | 3831.81(3638.31-4027.96) | 6078.52(5771.56-6389.67) |  | 8859.66(7997.98-9712.28) | 5527.25(4989.68-6059.18) |  | -0.40(-0.50--0.30) |
|  | Female |  | 5771.87(5367.43-6181.90) | 315.04(292.97-337.42) |  | 12045.17(10877.31-13342.69) | 498.66(450.31-552.37) |  | 1.60(1.50-1.70) |
|  |  | 5-14 years | 4.50(3.86-5.30) | 1.31(1.13-1.55) |  | 4.01(3.43-4.67) | 1.25(1.07-1.46) |  | -0.20(-0.70-0.30) |
|  |  | 15-49 years | 1040.47(935.23-1165.24) | 109.71(98.61-122.86) |  | 1373.02(1197.37-1567.02) | 113.10(98.63-129.08) |  | 0.20(-0.20-0.60) |
|  |  | 50-69 years | 3141.25(2915.67-3369.68) | 1192.85(1107.19-1279.59) |  | 6261.16(5653.72-6983.80) | 1204.52(1087.66-1343.54) |  | 0.00(-0.10-0.10) |
|  |  | ≥70 years | 1585.65(1478.99-1674.09) | 1667.05(1554.91-1760.03) |  | 4406.99(3856.89-4850.29) | 2128.50(1862.81-2342.60) |  | 0.800.800.90 |

ASDR, age standardized DALYs rate; AAPC, Annual percent change; UI, uncertainty interval.
